# CLOCK and BMAL1 stabilize and activate RHOA to promote F-actin formation in cancer cells

**DOI:** 10.1038/s12276-018-0156-4

**Published:** 2018-10-04

**Authors:** Teng-jiao Ma, Zhi-wei Zhang, Yi-lu Lu, Ying-ying Zhang, Da-chang Tao, Yun-qiang Liu, Yong-xin Ma

**Affiliations:** Department of Medical Genetics, State Key Laboratory of Biotherapy, West China Hospital and Collaborative Innovation Center, Sichuan University, 610041 Chengdu, China

**Keywords:** RHO signalling, Cancer of unknown primary

## Abstract

Circadian genes control most of the physiological functions in cancer cells, including cell proliferation, migration, and invasion. The CLOCK and BMAL1 complex plays a central role in circadian rhythms. Previous studies have shown that circadian genes may act as oncogenes or tumor-suppressor genes. In addition, F-actin, regulated by RHOA, has been shown to participate in tumor progression. However, the roles of the *CLOCK* and *BMAL1* genes in the regulation of tumor progression via the RHOA-ROCK-CFL pathway remain largely unclear. Here we first indicate that the rearrangement of F-actin is regulated by CLOCK and BMAL1. We found that CLOCK and BMAL1 can upregulate RHOA expression by inhibiting CUL3-mediated ubiquitination and activate RHOA by reducing the interaction between RHOA and RhoGDI. Consequently, CLOCK and BMAL1 control the expression of the components of the RHOA-ROCK-CFL pathway, which alters the dynamics of F-actin/G-actin turnover and promotes cancer cell proliferation, migration, and invasion. In conclusion, our research proposes a novel insight into the role of CLOCK and BMAL1 in tumor cells.

## Cancer: Clocking the triggers for tumor progression

Two proteins involved in regulating circadian rhythms may promote cancer tumor growth by changing the internal structures of cells. Circadian clock proteins play key roles in cell division, metabolism, and immune responses. Certain clock proteins are implicated in uncontrolled cell and tumor growth. Yong-xin Ma at Sichuan University in Chengdu, China and co-workers have shown that two clock proteins, CLOCK and BMAL1, regulate the arrangement of F-actin microfilaments in the cytoskeleton of cells. The researchers found that CLOCK and BMAL1 interact with an enzyme that promotes the formation of cellular fibers and regulates cell shape and motility. The overexpression of these clock proteins in cancers increases the levels of this enzyme, which changes F-actin structures inside cells. This promotes cancer cell proliferation, migration, and invasion.

## Introduction

The circadian rhythm, a ubiquitous mechanism, enables organisms to maintain temporal coordination between endogenous biological processes and the ambient environment^1^. Circadian clocks display oscillations with a periodicity of almost 24 h that matches the day–night cycle and can be found in most bodily tissues. These clocks control a wide variety of biological processes in organisms, including two hallmarks of cancer: cell division and metabolism^2^. Research has shown that the disruption of circadian timekeeping is associated with uncontrolled cell growth and cancer^3,4^. Additionally, circadian genes have also been shown to interact with oncogenes and tumor-suppressor genes in tumorigenesis^5^. In mammals, the molecular mechanism of the biological clock is based on transcriptional/translational autoregulatory feedback loops, which are composed of a set of clock genes. Two transcription factors, CLOCK (Circadian Locomoter Output Cycles Kaput) and BMAL1 (Brain and Muscle ARNT-Like 1), play a core role in this feedback system, working as one heterodimer^6^. Additionally, there is evidence^7,8^ showing that both oncogenes and tumor-suppressor genes are regulated by CLOCK and BMAL1 in tumor cells, which indicates regulatory roles of those two proteins in cancers.

The RHO family, a group of small GTPases, participates in the mediation of multiple processes of tumor progression, including the processes of cell transformation, cytokinesis, angiogenesis, extracellular matrix deposition, and tumor cell dissemination^9^. RHOA (Ras Homolog Family Member A), a member of the RHO family, promotes the formation of stress fibers and focal adhesions through actin–myosin contractility control, thereby regulating cell shape, attachment, and motility^10,11^. Like many other RHO family members, the function of RHOA is regulated by GEFs (guanine nucleotide exchange factors), GAPs (GTPase-activating proteins), and GDIs (guanine nucleotide dissociation inhibitors). GEFs catalyze GDP-to-GTP exchange (activation), while GAPs stimulate GTP hydrolysis (inactivation). GDIs sequester RHOA in the cytoplasm, preventing its further interaction with other downstream effectors^12^. As a downstream effector of RHOA, ROCK (Rho-associated coiled-coil containing kinase) plays vital roles in facilitating actomyosin cytoskeleton contractility^13^. Activated ROCK promotes actin organization by phosphorylating several downstream target proteins during mitosis, including actin-depolymerizing factor CFL (cofilin), MLC (myosin light chain), and LIM kinase^14^. When phosphorylated by the RHOA-ROCK pathway, CFL is inactivated, leading to polymerization of G-actin into F-actin^15,16^. This process can directly affect the formation of lamellipodium in cancer cells, which plays a vital role in cancer metastasis^17^.

Although accumulating evidence has indicated critical roles of circadian rhythms and RHO family proteins, whether there is crosstalk between those two systems in tumor cells is still unclear. In this study, we demonstrate for the first time that CLOCK and BMAL1 promote cytoskeletal F-actin filament formation by regulating the RHOA-ROCK-CFL pathway, revealing a novel mechanism of circadian genes in tumor cells.

## Materials and methods

### Cell culture and transfection

HeLa and HepG2 cells used in this research were conserved in our laboratory as previously described^18,19^. They were grown in Dulbecco’s modified Eagle’s medium with 5% fetal bovine serum (FBS, HyClone, USA) cultured in a humidified incubator (at 37 °C, 5% CO_2_) before transfection was performed, and the medium was changed every other day. According to the manufacturer’s protocols, all transfections were performed using the transfection reagent (#114–15, jetPRIME, France) in six-well plates.

### Reagents and reagent kits

Cycloheximide (CHX) treatment was performed 48 h after transfection, with a final concentration of 20 μg/ml for an indicated period of time, and cells in specific groups were treated with MG132 (20 μM), a proteasome inhibitor, for 6 h. After that, cells were harvested and analyzed by western blotting (WB) using appropriate antibodies.

For the cytosolic (C)/nuclear (N) fractionation assay, the Nuclear and Cytoplasmic Protein Extraction Kit (P0027, Beyotime, China) was used. For the cytosolic/membrane fractionation assay, the Membrane and Cytosol Protein Extraction Kit (P0033, Beyotime, China) was purchased. All experiments were repeated at least three times unless stated otherwise.

### Expression plasmids, short hairpin RNAs (shRNAs), and antibodies

DNA fragments containing the open reading frame encoding FLAG-tagged RHOA, HA-tagged CLOCK, and HA-tagged BMAL1 were constructed and inserted into pcDNA3.1(+) expression plasmids (Invitrogen, Waltham, USA); FLAG-tagged CUL3 and FLAG-tagged RhoGDI were constructed and inserted into pENTER expression plasmids (Vigene Biosciences, Jinan, China); shRNAs for BMAL1, CLOCK, RHOA, and CUL3 were constructed and cloned into pGPU6/GFP/Neo (GenePharma, Shanghai, China). The target sequences for shRNAs were as follows: BMAL1 shRNA (shBMAL1): 5′-CUAUGAGAUUCCUCAACUACAGAAG-3′; CLOCK shRNA (shCLOCK): 5′-AATGGGAAACGCTACTTCGAA-3′; RHOA shRNA (shRHOA): 5′-GGAAAGAGCUGCAUAUUAATT-3′; NC shRNA (shNC): 5′-UUCUCCGAACGUGUCACGUTT-3′; siRNA (siCUL3 #1): 5′-GCGAGAAGAUGUACUAAAUTT-3′; siRNA (siCUL3 #2): 5′-GGCAAACUCUAUUGGAUAUTT-3′; and siRNA (siCUL3 #3): 5′- GACCGUCUCUUUAAACAAATT-3′.

The antibodies used in the WB and coimmunoprecipitation (Co-IP) experiments are listed below: mouse monoclonal anti-FLAG antibody and mouse monoclonal anti-HA antibody were from Zen BioScience (Zen BioScience, Chengdu, China). Goat polyclonal anti-CLOCK antibody and goat polyclonal anti-BMAL1 antibody were purchased from Cell Signaling Technology (CST, Danvers, USA). Mouse polyclonal anti-RHOA antibody, mouse monoclonal anti-Rho GDI antibody, rabbit polyclonal anti-HA antibody, mouse monoclonal anti-ROCK2 antibody, rabbit polyclonal anti-ROCK1, mouse monoclonal anti-CFL antibody, rabbit polyclonal anti-p-CFL (Ser3) antibody, and mouse monoclonal anti-CUL3 antibody were purchased from Santa Cruz Biotechnology (Santa Cruz, USA). Mouse monoclonal anti-GAPDH was purchased from Abcam (Abcam, Cambridge, UK).

### Co-IP and WB

Universal protein extraction buffer (#PP1801, Bioteke, China) with protease inhibitor (#04693116001, Roche, Switzerland) was used to lyse cells transfected with designated plasmids for 48 h. The lysate was subjected to IP with protein A + G agarose beads (#P2012, Beyotime, China) and specified antibodies. Co-IP proteins were further separated by sodium dodecyl sulfate-polyacrylamide gel electrophoresis (SDS-PAGE) and detected using corresponding primary antibodies and secondary antibodies conjugated to horseradish peroxidase (HRP), after which the proteins were transferred to polyvinylidene difluoride membranes (#IPVH00010, Millipore, USA). Proteins were visualized using an Immobile Western Chemiluminescence HRP Substrate Kit (#WBKLS0100, Millipore, USA).

### RHOA activity assay

Activated RHOA was pulled down using the GST-rhotekin-Rho-binding domain (GST-RBD) that specifically binds activated RHOA (#BK036-S, cytoskeleton, USA). In brief, cells stimulated with agonists or GTPγS for the indicated durations were washed twice with phosphate-buffered saline (PBS), lysed with 500 μl cell lysis buffer, and then centrifuged at 10,000 rpm for 5 min. Equal volumes of supernatants were incubated with Rhotekin-RBD affinity beads for 1 h at 4 °C, followed by two washes in lysis buffer and three washes in the supplied wash buffer. Bound proteins were eluted in 3× 1% SDS sample buffer and examined by 10% SDS-PAGE and WB analysis using the anti-RHOA antibody.

### Immunofluorescence staining

Cells were first fixed with 4% paraformaldehyde (#P1110, Solarbio, USA) in PBS for 10–15 min and permeabilized with 0.5% Triton X-100 (#T8200, Solarbio, USA) for 5 min. Then the cells were blocked with 1% bovine serum albumin (#A8010, Solarbio, USA) for 30–45 min. Fixed cells were incubated overnight at 4 °C with specific primary antibodies. Finally, the cells were incubated with Alexa Fluor® 350 (#A10039, Thermo Fisher Scientific, USA), Alexa Fluor® 555 (#A-31570, Thermo Fisher Scientific, USA), and Alexa Fluor® 488 (#A-11055, Thermo Fisher Scientific, USA) for 2 h at room temperature. Each step was followed by three 5-min washes in PBS. DAPI (4, 6-diamidino-2-phenylindole; #D9542, Sigma, USA) was used for nuclear staining. Fluorescent images were obtained using a confocal microscope (Olympus, Japan).

For F-action staining, cells were fixed with 4% paraformaldehyde and permeabilized with 0.5% Triton X-100. The cells were then incubated with 5 μg/ml phalloidin for 1 h in the dark. Then DAPI was used for nuclear staining.

### Quantitative PCR

Quantitative PCR was performed with a CFX96 Touch real-time PCR detection system (Bio-Rad, USA) using SYBR green PCR Mastermix (#SR1110, Solarbio, USA). The mean values of the results were analyzed and normalized against the housekeeping gene *β-actin*.

### In vitro binding assays

The TNT® Quick Coupled in Vitro Transcription/Translation System (#REFL1171, Promega, USA) was used for protein interaction analysis in vitro with circular plasmid DNAs containing the T7 promoter. The CLOCK-expressing plasmid (CLOCK-pcDNA3.1-HA), BMAL1-expressing plasmid (BMAL1-pcDNA3.1-HA), RhoGDI-expressing plasmid (RhoGDI-pENTER-FLAG), and RHOA-expressing plasmid (RHOA-pcDNA3.1-FLAG) were used in this research. In vitro protein coexpression assays for CLOCK/BMAL1/RHOA, CLOCK/RhoGDI/RHOA, and RhoGDI/BMAL1/RHOA were carried out separately in three reactions using the TNT® system. One microgram of total plasmid DNA was diluted in 50 μl and incubated at 30 °C for 1.5 h. Then 2 μl of each product was used to detect the protein expression of CLOCK/BMAL1 and RHOA by WB with specific antibodies. Subsequently, 30 μl of each translated protein was mixed together in 200 μl lysis buffer for Co-IP assays.

### Actin segmentation by ultracentrifugation

Cells were lysed directly in the dish using RIPA lysate (#P0013B, Beyotime, China) with 1% phenylmethanesulfonylfluoride (#ST506, Beyotime, China) for 30 min on ice before being collected into 1.5 ml Eppendorf tubes. Then the cell lysates were separated by ultracentrifugation at 15,000 × *g* for 30 min at 4 °C. The supernatant containing G-actin was removed to a fresh tube, while the pellet containing F-actin was resuspended in cold 1× PBS before being ultracentrifuged at 15,000 × *g* for 5 min twice. After centrifugation, the pellet was resuspended in F-actin extracting solution (1.5 mM guanidine hydrochloride, 1 mM sodium acetate, 1 mM CaCl_2_, 1 mM ATP, 20 mM Tris-HCl, pH 7.5) for 1 h on ice to dissolve F-actin before being ultracentrifuged at 15,000 × *g* for 30 min at 4 °C. Then both F-actin and G-actin became β-actin monomers, which was detected using an antibody against β-actin by WB.

### Cell proliferation assay

Cell proliferation was performed using a WST-8 Cell Counting Kit-8 (#C0038, Beyotime, China) according to the manufacturer’s instructions. The absorbance at 450 nm was measured using a microplate reader (BioTek, VT, USA). The relative proliferation ratio was calculated by absorbance and normalized to the absorbance of a control that was given an arbitrary value of one.

### Cell migration and invasion assays

For the cell migration assay, the appropriate number of cells for 100% confluence in 24 h was plated in a 6-well plate. We pressed a 200-μl pipette tip firmly against the top of the tissue culture plate and swiftly made a vertical wound through the cell monolayer in the biosafety hood. Snapshot pictures were analyzed by measuring the distance from one side of the wound to the other to clearly show wound closure over time using a scatter plot or bar graph. A fluorescence inverse microscope (Olympus, Japan) was used to capture the images. For the cell invasion assay, transfected cells in serum-free medium were plated in a Biocoat Matrigel invasion chamber with 5% FBS in media and incubated overnight. Finally, cells were fixed in methanol for 10 min and stained with Wright–Giemsa (Jiancheng Biotech, Nanjing, China).

### Data availability

The data shown in Fig. 8a are available on the *Oncomine* database, http://www.oncomine.org.

### Statistical analysis

All experiments were repeated three times. WB results were quantified with the ImageJ software. Data are presented as the mean ± sd. Statistical analysis was performed by applying GraphPad Prism (version 5.0) and Student’s *t-*test. Values of *p* < 0.05 were considered significant and indicated by asterisks in the figures.

## Results

### CLOCK and BMAL1 stimulate the formation of F-actin

To determine the functional role of CLOCK and BMAL1 in tumor cells, two types of shRNAs (shCLOCK and shBMAL1 for short) were used to attenuate CLOCK and BMAL1 expression, and the CLOCK-expressing plasmid CLOCK-pcDNA3.1-HA and the BMAL1-expressing plasmid BMAL1-pcDNA3.1-HA (CLOCK+ and BMAL1+ for short) were used to increase their levels.

**Fig. 1:**
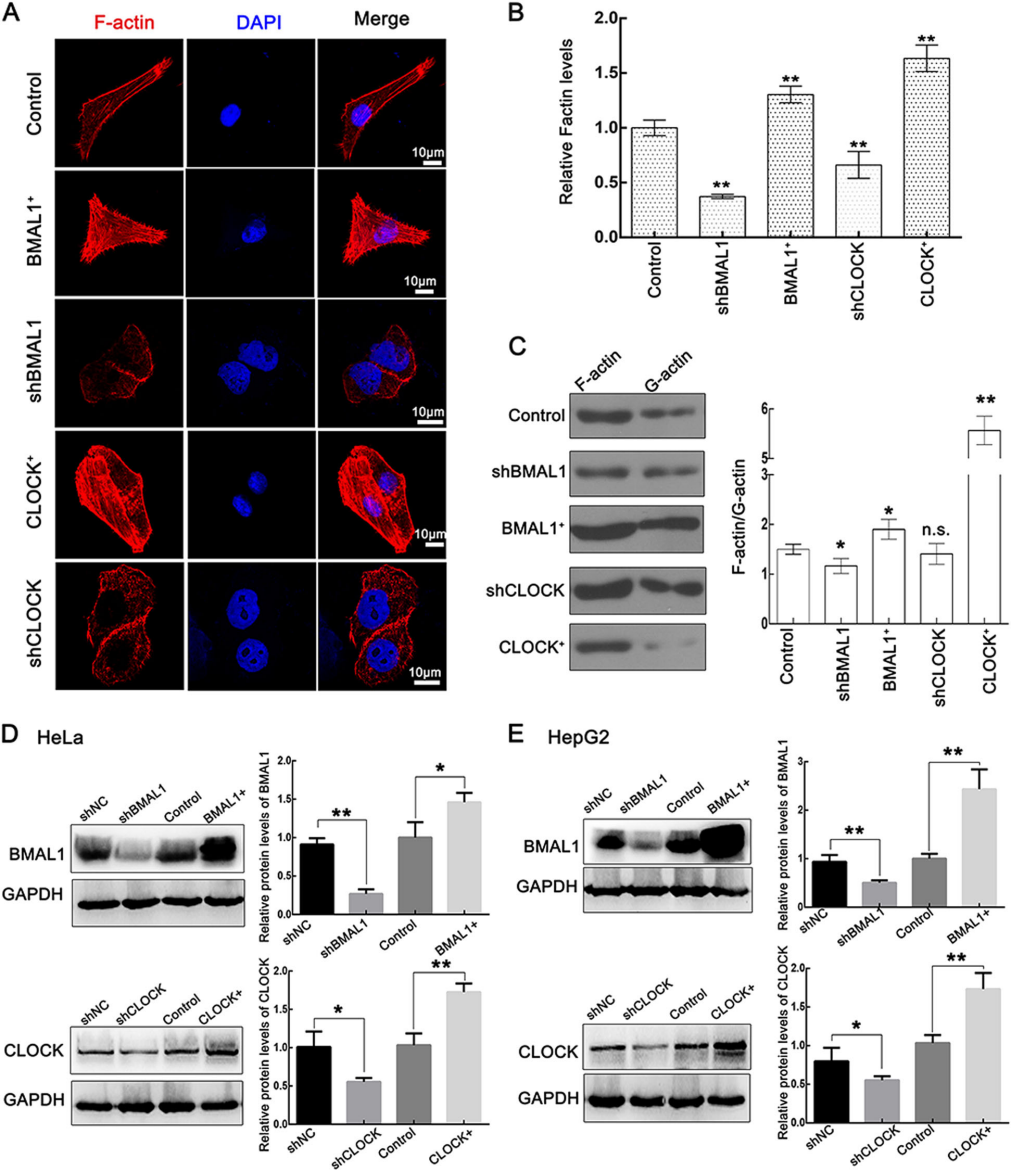
CLOCK and BMAL1 enhance the formation of F-actin. **a**, **b** F-actin was stained with TRITC-phalloidin (Red) in HepG2 cells transfected with HA-tagged BMAL1 (BMAL1+), HA-tagged CLOCK (CLOCK+), shRNA for BMAL1 (shBMAL1), and CLOCK (shCLOCK) vectors. Nuclei were stained with DAPI. The scale bars represent 10 μm. The F-actin levels were quantified with the fluorescence intensity ratio using ImageJ. The fluorescence intensity of the control was taken as 1. Data were analyzed using a *t* test (*n* = 3) and presented as the mean ± sd; *p < 0.05; **p < 0.01. **c** F-actin and G-actin from control, shBMAL1, BMAL1+, shCLOCK, and CLOCK+ HeLa cells were separated by ultraspeed centrifugation and analyzed by western blotting using an antibody against β-actin. The mean ratio of F-actin to G-actin and SD were plotted, and a *t* test was performed (*n* = 3); n.s. not significant; *p < 0.05; **p < 0.01. **d**, **e** The expression levels of CLOCK and BMAL1 were verified by western blotting in HeLa and HepG2 cells transfected with the corresponding plasmids, respectively. GAPDH was used as a loading control, and the protein level of the control was taken as 1. Data were analyzed using a *t* test (*n* = 3) and presented as the mean ± sd; *p < 0.05; **p < 0.01

Characterization of CLOCK and BMAL1 overexpression in HepG2 cells by fluorescent phalloidin staining showed that actin filaments were elongated and thus became more prominent. Meanwhile, the stress fibers observed in CLOCK knockdown and BMAL1 knockdown cells were apparently shortened and weakened (Fig. 1a, b). Moreover, the F-actin and G-actin extraction assays were conducted to measure the F-actin turnover. The CLOCK+ and BMAL1+ groups in HeLa cells had a significantly higher F-actin to G-actin ratio than the group, while the shBMAL1 group had a significantly lower ratio of F-actin to G-actin than the control group (Fig. 1c). These findings suggest that CLOCK and BMAL1 may play key roles in the formation of the actin cytoskeleton.

CLOCK and BMAL1 abundance were verified by WB using antibodies that recognize CLOCK or BMAL1 in HeLa and HepG2 cells, respectively (Fig. 1d, e). The shNC plasmid and pcDNA3.1(+) vector were used as controls for knockdown and overexpression assays, respectively, and only the pcDNA3.1(+) vector was used as the control in subsequent experiments, as no differences were detected between the shNC plasmid and pcDNA3.1(+) vector-transfected groups.

### CLOCK and BMAL1 interact with RHOA

**Fig. 2:**
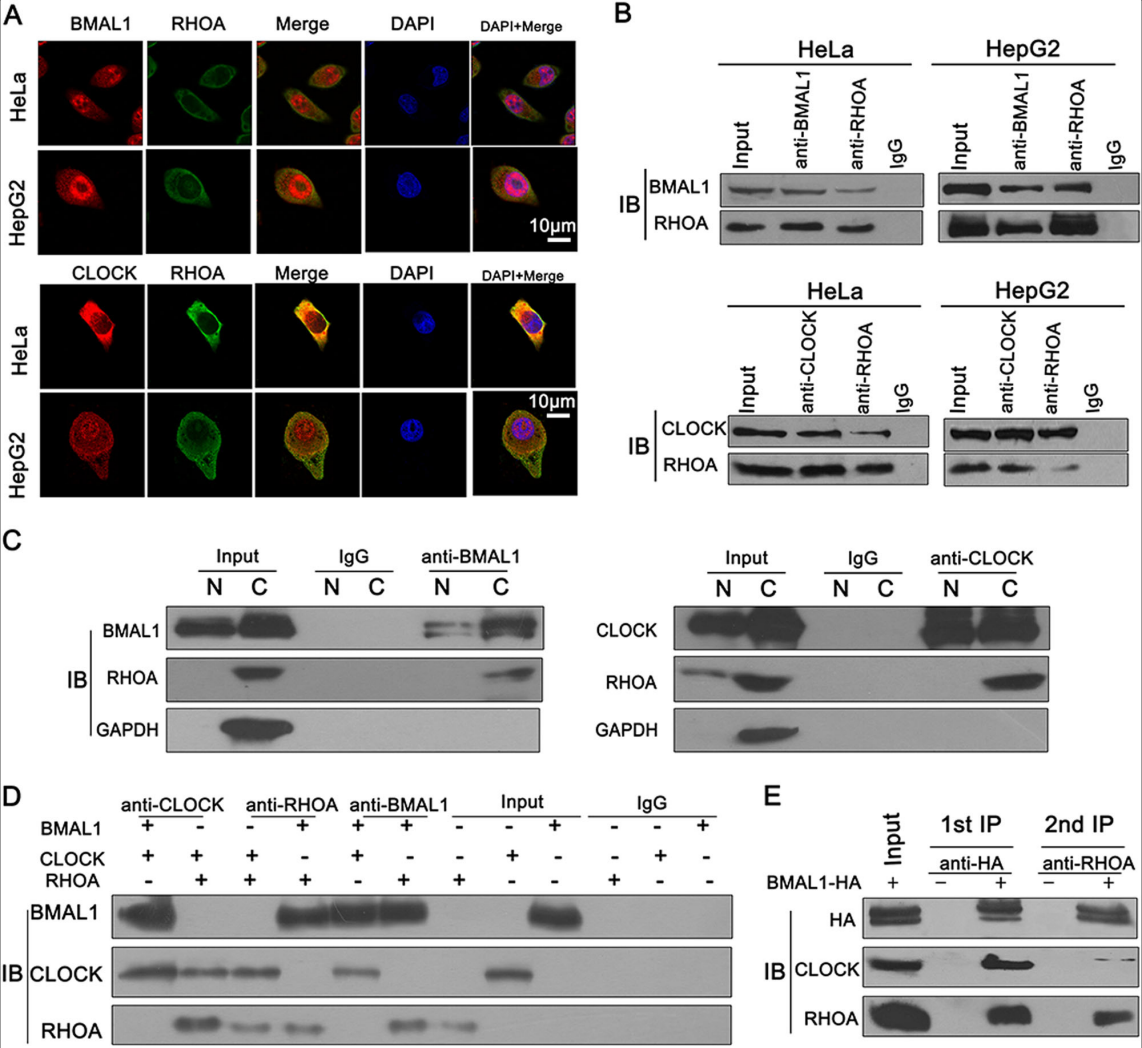
CLOCK and BMAL1 interact with RHOA. **a** Immunofluorescent staining of endogenous BMAL1/CLOCK (red) and RHOA (green) in HeLa and HepG2 cells. Blue color represents the nucleus stained with DAPI. The scale bars represent 10 μm. **b** The complex was immunoprecipitated from total protein extracts collected from HeLa and HepG2 cells. The immunoprecipitates were analyzed by western blot with antibodies against BMAL1, CLOCK, or RHOA. **c** Cytosolic (C) and nuclear (N) fractionation followed by Co-IP were performed in HeLa cells. **d** The TNT® Quick Coupled Transcription/Translation System was used for protein expression in vitro. The synthesized proteins were then analyzed by Co-IP with antibodies against BMAL1, CLOCK, or RHOA. **e** Two-step IP assay. Cell lysates were immunoprecipitated with the anti-HA antibody, and then BMAL1-HA and the associated proteins were eluted with 4 × HA peptide (30 μmol/μl)

To further investigate the roles of the circadian core protein CLOCK and BMAL1 in tumor cells, we performed immunofluorescence assays and found that both endogenous CLOCK (red) and BMAL1 (red) overlapped with RHOA (green), mainly in the cytoplasm (Fig. 2a). Next, Co-IP assays were performed to investigate the physiologically relevant protein–protein interactions, and the results showed that both endogenous CLOCK and BMAL1 can bind with RHOA in HeLa and HepG2 cells (Fig. 2b). The Co-IP assays using the cytosolic (C) and nuclear (N) fractions further determined that CLOCK and BMAL1 bind with RHOA mostly in the cytoplasm (Fig. 2c). To investigate whether BMAL1, CLOCK, and RHOA can form a complex, immunofluorescence assays were conducted, and the results showed that BMAL1, CLOCK, and RHOA were colocalized mostly in the cytoplasm (Fig. S1).

Next, to examine the direct interaction among BMAL1, CLOCK, and RHOA, the TNT^®^ Quick Coupled Transcription/Translation (TNT) System was used for protein expression in vitro. Coexpressed BMAL, CLOCK, and RHOA were then subjected to IP. The results indicated that BMAL1, CLOCK, and RHOA can directly bind to each other in vitro (Fig. 2d), suggesting that BMAL1, CLOCK, and RHOA can form a complex. Taken together, these results led to the speculation that BMAL1, CLOCK, and RHOA could form a complex, and a two-step IP assay was performed to verify this hypothesis (Fig. 2e).

### CLOCK and BMAL1 upregulate RHOA at the protein level

**Fig. 3:**
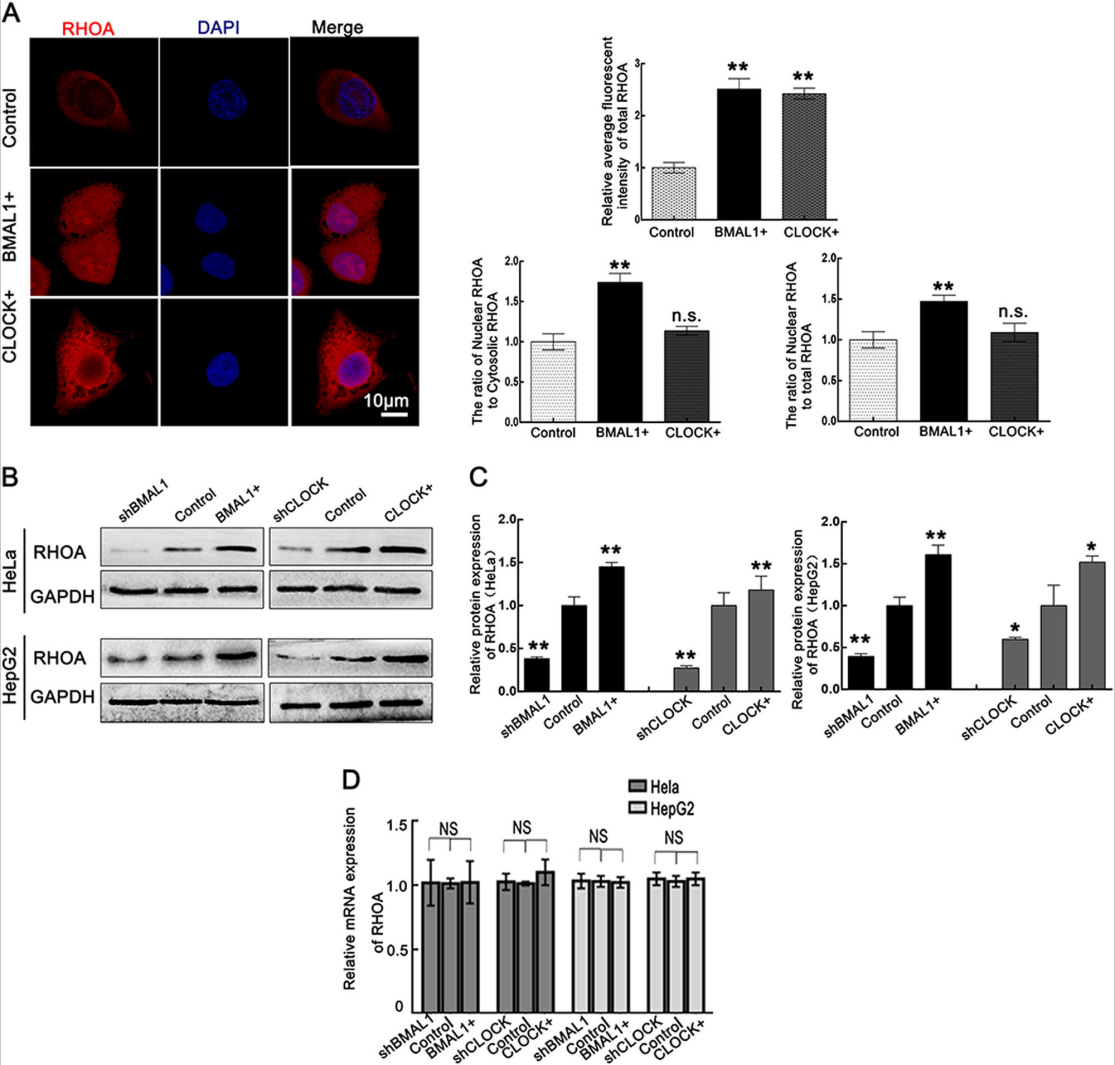
CLOCK and BMAL1 upregulate the expression of RHOA protein. **a** Immunofluorescent staining of RHOA (Red) in BMAL1+, control, and CLOCK+ HeLa cells (left panel). Nuclei were stained with DAPI. The scale bars represent 10 μm. The quantification of RHOA was performed with ImageJ and analyzed (right panel) **p < 0.01. **b**, **c** Expression of RHOA at the protein level was examined using western blotting in shBMAL1, BMAL1+, shCLOCK, and CLOCK+ HeLa cells. RHOA expression was quantified with ImageJ and then normalized to GAPDH expression. The protein level of the control was taken as 1. Data were analyzed using a *t* test (*n* = 3) and presented as the mean ± sd; *p < 0.05; **p < 0.01. **d** Expression of RHOA at the mRNA level was examined by qPCR in shBMAL1, BMAL1+, shCLOCK, and CLOCK+ cells. Data are presented as the mean ± sd (*n* = 3); n.s. not significant

As we have shown that both CLOCK and BMAL1 can interact with RHOA, we then sought to determine whether CLOCK or BMAL1 regulate the expression of RHOA. Immunofluorescent staining showed that overexpression of CLOCK or BMAL1 increased the amount of RHOA (red) in HeLa cells. In control and CLOCK-overexpressing cells, RHOA was mainly expressed in the cytosol. Interestingly, when BMAL1 was overexpressed, RHOA was localized in both the cytosol and nucleus, suggesting that BMAL1 can regulate RHOA localization as well as its expression (Fig. 3a). Additionally, WB results showed that overexpression of CLOCK and BMAL1 upregulated RHOA expression at the protein level in HeLa and HepG2 cells. Meanwhile, CLOCK and BMAL1 knockdown inhibited RHOA expression at the protein level in HeLa and HepG2 cells (Fig. 3b, c). Then quantitative real-time PCR was performed to detect the expression level of RHOA mRNA in HeLa and HepG2 cells, and no significant changes in RHOA mRNA levels were detected after BMAL1 or CLOCK expression was altered (Fig. 3d).

### CLOCK and BMAL1 block CUL3-mediated ubiquitination and degradation of RHOA

**Fig. 4:**
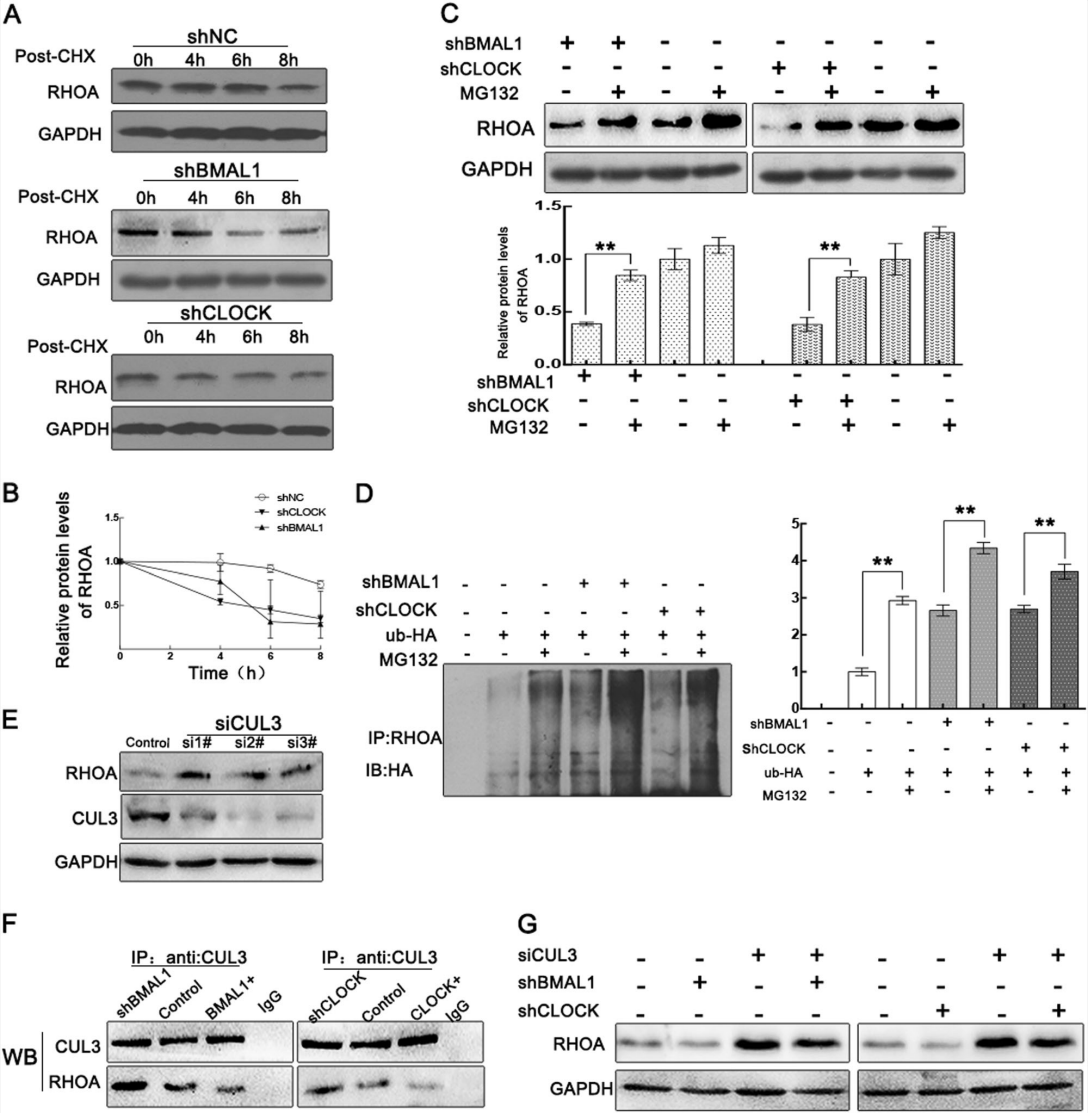
CLOCK and BMAL1 block CUL3-mediated RHOA ubiquitin degradation. **a**, **b** Stability analysis of RHOA protein. **a** HeLa and HepG2 cells transfected with shNC, shBMAL1, or shCLOCK were treated with cycloheximide (20 μg/ml) for the indicated duration before harvesting. Expression of RHOA was detected by western blotting. **b** RHOA expression was quantified with ImageJ and then normalized to GAPDH expression. The protein level at 0 h was taken as 1. **c** Cells were treated as indicated, and the concentration of MG132 used was 20 μM. Data are presented as the mean ± sd (*n* = 3); *p < 0.05; **p < 0.01; n.s. not significant. **d** The ubiquitination levels of RHOA were analyzed by Co-IP assay. HeLa cells transfected with HA-ubiquitin (Ub) were treated with 20 μM MG132 for 6 h before harvesting. Data are presented as the mean ± sd (*n* = 3); *p < 0.05; **p < 0.01; n.s. not significant. **e** The efficiency of siCUL3 was tested with a western blot assay. siCUL3 #2 siRNA was selected for later experiments. **f** The complex was immunoprecipitated from total protein extracts collected from HeLa cells transfected with corresponding plasmids. The proteins were then analyzed by Co-IP with antibodies against CUL3. **g** The expression of RHOA was verified by western blotting in HeLa cells transfected with corresponding plasmids

The results above indicate that neither CLOCK nor BMAL1 affect RHOA mRNA expression, which implies that CLOCK and BMAL1 may regulate RHOA expression by affecting protein degradation. We then measured RHOA protein stability in cells transfected with CLOCK or BMAL1 shRNA vectors and treated with 20 μg/ml CHX to inhibit protein synthesis. WB results showed that CLOCK and BMAL1 knockdown led to a significant increase in RHOA degradation (Fig. 4a, b). The downregulation of RHOA by BMAL1 or CLOCK knockdown was restored by treatment with the proteasome inhibitor MG132, indicating that CLOCK and BMAL1 can inhibit the proteasome-mediated degradation of RHOA (Fig. 4c). Additionally, a ubiquitination assay was performed, illustrating an increase in RHOA ubiquitination levels induced by CLOCK and BMAL1 knockdown (Fig. 4d).

Previous literature^20–22^ reported that the E3 ubiquitin ligase CUL3, a cullin family scaffold protein, can affect the stability of the RHOA protein and induce RHOA ubiquitin-mediated proteolysis. Therefore, we assumed that CLOCK and BMAL1 may suppress the ubiquitination of RHOA that is induced by CUL3. First, we measured the increased RHOA protein abundance induced by CUL3 knockdown (Fig. 4e). We further confirmed that this interaction between CUL3 and RHOA was potentiated in CLOCK- and BMAL1-knockdown cells and decreased in CLOCK- and BMAL1-overexpressing cells, indicating that CLOCK and BMAL1 compete with CUL3 in their interaction with RHOA (Fig. 4f). Additionally, CUL3 knockdown restored the decrease in RHOA induced by CLOCK and BMAL1 (Fig. 4g). These results indicated that CLOCK and BMAL1 can inhibit the ubiquitin-mediated degradation of RHOA by suppressing the interaction between CUL3 and RHOA.

### CLOCK and BMAL1 block the interaction between RHOA and GDI to activate the RHOA-ROCK-CFL pathway

**Fig. 5:**
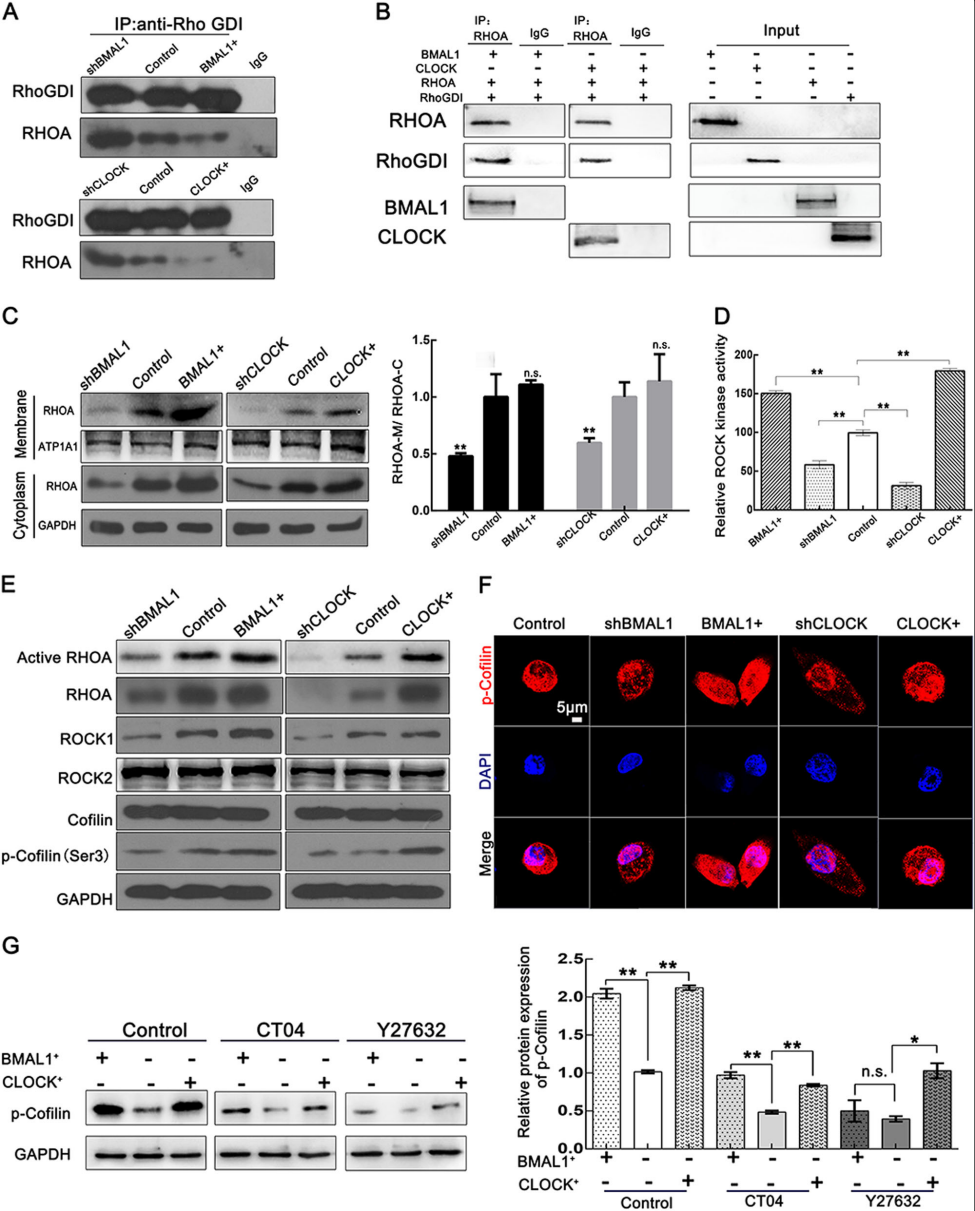
CLOCK and BMAL1 regulate the RHOA-ROCK-CFL pathway. **a** The complex was immunoprecipitated from total protein extracts collected from HeLa cells transfected with corresponding plasmids. The proteins were then analyzed by Co-IP with antibodies against RhoGDI. **b** The TNT® Quick Coupled Transcription/Translation System was used here for protein (BMAL1, CLOCK, RHOA, and RHOGDI) synthesis. The synthesized proteins were then analyzed by Co-IP with corresponding antibodies. **c** The RHOA distribution was affected by BMAL1 or CLOCK, as detected by a cytosolic/membrane fractionation assay in HeLa cells. *p < 0.05; **p < 0.01, n.s. no significant. **d** HeLa cells transfected with the indicated vectors were harvested and analyzed using a 96-well ROCK Activity Assay Kit. The activity levels were normalized to those in the control, which was given an arbitrary value of 1 **p < 0.01. **e** CLOCK and BMAL1 can promote the activation of RHOA and the expression of RHOA and other proteins involved in the RHOA-ROCK-CFL pathway. Activated RHOA was pulled down using a GST-Rhotekin-Rho-binding domain (RBD) fusion protein. **f** Immunofluorescent staining of p-cofilin (Ser-3) in HeLa cells transfected with BMAL1+, CLOCK+, shBMAL1, and shCLOCK. Scale bar, 5 µm. **g** HeLa cells transfected with the indicated plasmids were treated with 1 μg/ml CT04 (RHOA inhibitor) for 2 h and Y27632 (ROCK inhibitor) for 20 h before whole-cell lysates were extracted and analyzed. Statistical results of the p-cofilin protein level are shown to the right *p < 0.05; **p < 0.01, n.s. no significant

Previous research has shown that RhoGDIs can inactivate RHOA, resulting in the passive localization of RHOA to the cytoplasm, where it is sequestered and its activation or interaction with effectors are prevented^23,24^. To further prove whether CLOCK and BMAL1 can regulate the interaction between GDI and RHOA, Co-IP assays were employed, and the results revealed that CLOCK and BMAL1 reduced the interaction between RHOA and RhoGDI (Fig. 5a). Both CLOCK and BMAL1 are indicated to be able to compete with RhoGDI toward RHOA in cells. To investigate the direct combination in vitro when CLOCK/RhoGDI/RHOA and BMAL1/RhoGDI/RHOA are coexpressed, we synthesized the proteins BMAL1, CLOCK, RHOA, and RHOGDI in vitro using the TNT® Quick Coupled Transcription/Translation System. The synthesized proteins were then analyzed by Co-IP with suitable antibodies. The results showed that RHOA bound directly to RHOGDI, CLOCK, or BMAL1 when they were coexpressed in vitro (Fig. 5b). The membrane (M)/cytosolic (C) fractionation assays further confirmed that knockdown of CLOCK and BMAL1 can attenuate the localization of RHOA on the cell membrane in HeLa cells (Fig. 5c). Notably, the effects of BMAL1 and CLOCK overexpression in this assay were both mild, potentially due to the intrinsically high endogenous expression of BMAL1 and CLOCK in cancer cells.

To investigate whether CLOCK and BMAL1 regulate ROCK activity as downstream effectors of RHOA, we then determined the ROCK kinase activity influenced by CLOCK and BMAL1 in HeLa cells. The results showed that ROCK kinase activity was significantly increased in CLOCK- and BMAL1-overexpressing cells and decreased in CLOCK- and BMAL1-knockdown cells (Fig. 5d). WB results further showed that overexpression of CLOCK and BMAL1 upregulated the activity of RHOA and the expression level of RHOA and its downstream proteins (ROCK and p-CFL) (Fig. 5e). The immunofluorescence assays further confirmed that overexpression of CLOCK and BMAL1 can enhance the level of Ser3-phosphorylated CFL (Fig. 5f).

The results above suggest that CLOCK and BMAL1 may regulate the RHOA-ROCK-CFL pathway. Next, Y27632 (the inhibitor of ROCK) and CT04 (the inhibitor of RHOA) were used to treat the cells, and the levels of phosphorylated CFL were tested. The results indicated that, in the presence of Y27632 or CT04, the effects of BMAL1 or CLOCK overexpression on CFL were attenuated (Fig. 5g).

### CLOCK and BMAL1 positively regulate F-actin filaments via RHOA

**Fig. 6:**
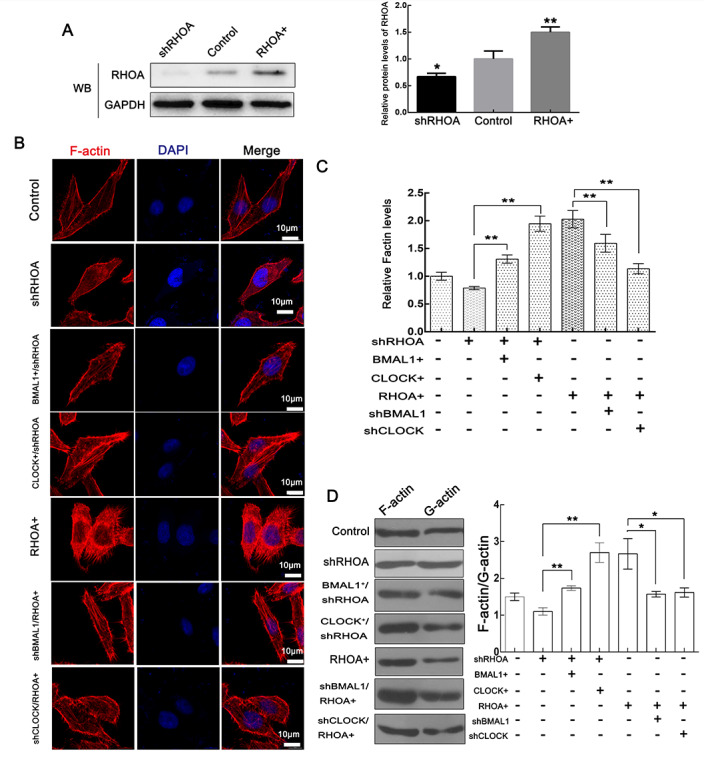
CLOCK and BMAL1 enhance F-actin filaments via the RHOA-ROCK-CFL pathway. **a** RHOA abundance was verified by western blotting in RHOA overexpression (RHOA+), RHOA knockdown (shRHOA), and control HeLa cells. GAPDH was used as a loading control. Data are presented as the mean ± sd (*n* = 3); *p < 0.05; **p < 0.01. **b**, **c** F-actin was stained with TRITC-phalloidin (Red) in HepG2 cells transfected with vectors. Nuclei were stained with DAPI. The scale bars represent 10 μm. The F-actin levels were quantified with the fluorescence intensity ratio using ImageJ. The fluorescence intensity of the control was taken as 1. Data were analyzed using a *t* test (*n* = 3) and presented as the mean ± sd; *p < 0.05; **p < 0.01. **d** F-actin and G-actin from HeLa cells were segmented by ultraspeed centrifugation and analyzed by western blotting using an antibody against β-actin. The data were quantified using ImageJ. *p < 0.05; **p < 0.01, n.s. not significant

CFL is the key F-actin regulator in spines, and its silencing leads to defects in spine morphology and function. The activity of CFL is tightly restricted by its phosphorylation (Ser3)^25–27^. As shown in Fig. 5, the overexpression of CLOCK and BMAL1 can promote the phosphorylation of CFL to downregulate its activity, thereby decreasing its ability to cut actin filaments and inhibiting the depolymerization of actin filaments. RHOA abundance was verified by WB using antibodies that recognize RHOA in HeLa cells (Fig. 6a). Immunofluorescence staining showed that overexpression of RHOA promoted a significant decrease in F-actin-to-G-actin ratio in response to CLOCK/BMAL1 knockdown. Meanwhile, RHOA knockdown significantly inhibited the increase in F actin-to-G-actin ratio in response to overexpression of CLOCK/BMAL1 (Fig. 6b, c). These results were confirmed by F-actin and G-actin extraction assays (Fig. 6d). Together, these findings revealed that CLOCK and BMAL1 regulate F-actin filament formation, at least partially, through the RHOA-ROCK-CFL pathway.

### CLOCK and BMAL1 can promote cell proliferation, migration, and invasion via RHOA

**Fig. 7:**
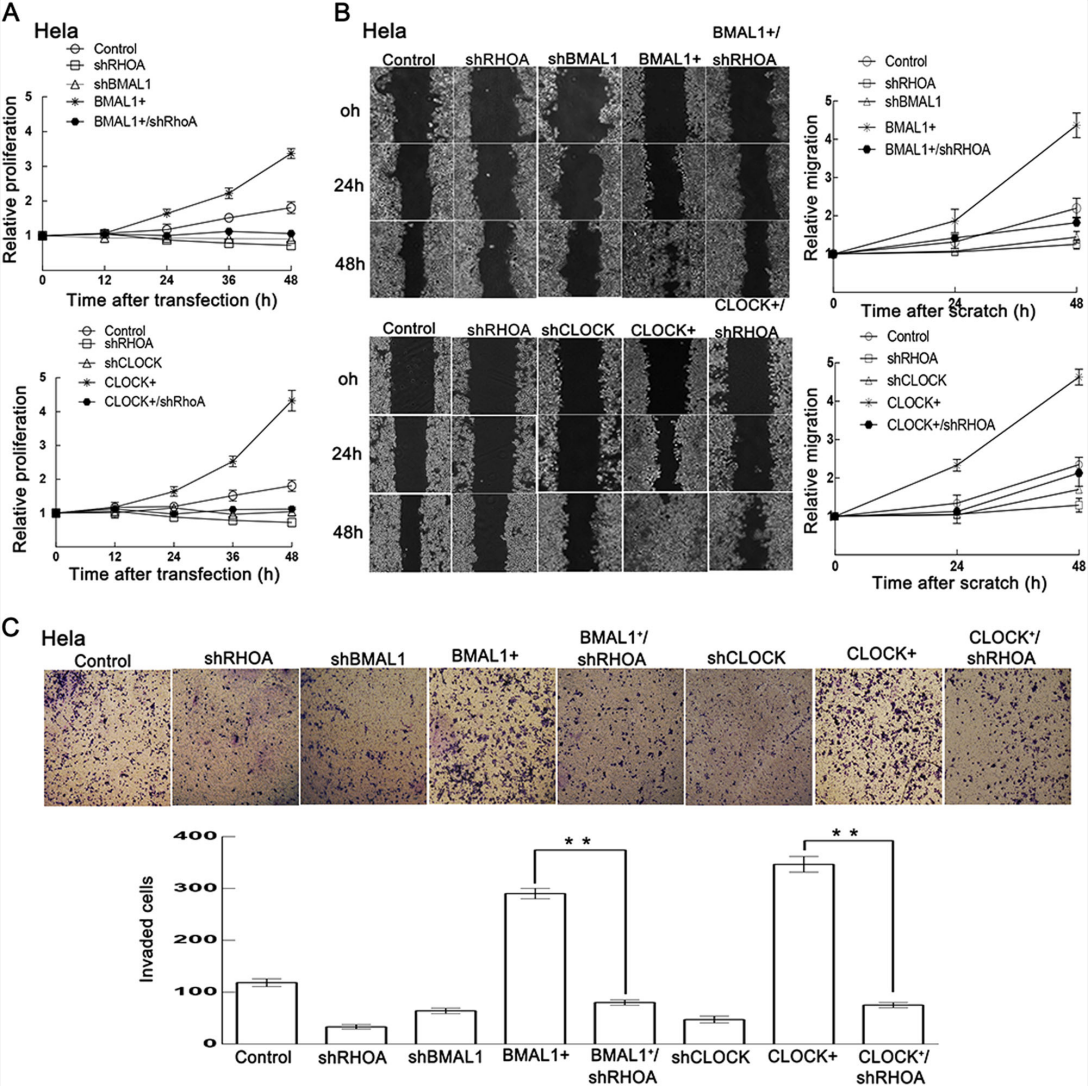
CLOCK and BMAL1 promote cell proliferation, migration, and invasion. **a** The cell viability of HeLa cells was determined by Cell Counting Kit-8 assay after plasmid transfection. The results were normalized to the control group (mean ± SD, *n* = 3). **b** Representative pictures of the scratch wound healing assay. During a 48-h wound closure assay, pictures were taken every 24 h using a time-lapse microscope. Representative pictures at 0, 24, and 48 h are shown. The distance of the wound was measured, and two line charts were used to display the width of the wound over time. Data were analyzed using a *t* test (*n* = 3) and presented as the mean ± sd. **c** Representative microscopic images of cells that migrated through the transwell in the migration assay. After cell migration and staining with crystal violet, pictures of the migrated cells (purple stained) were taken using a microscope. **p < 0.01

Cell proliferation, migration, and invasion are very useful and important for normal development, immune response, and disease processes, such as cancer metastasis and inflammation. Thus, to examine whether CLOCK and BMAL1 can promote tumor cell proliferation via RHOA, the Cell Counting Kit-8 assays were introduced to observe cell proliferation in HeLa cells. Enhanced cell proliferation induced by BMAL1 or CLOCK overexpression was observed and was attenuated by RHOA knockdown (Fig. 7a). Here we also examined tumor cell migration and invasion using two related assays to measure cancer metastasis. Scratch wound healing assay and transwell assay were used to analyze the ability of single cells to directionally respond to various chemoattractants. The results demonstrated that overexpression of CLOCK and BMAL1 markedly promoted cell migration and invasion, which was also attenuated by RHOA knockdown (Fig. 7b, c). These results suggested that CLOCK and BMAL1 can promote tumor cell migration and invasion through RHOA.

**Fig. 8:**
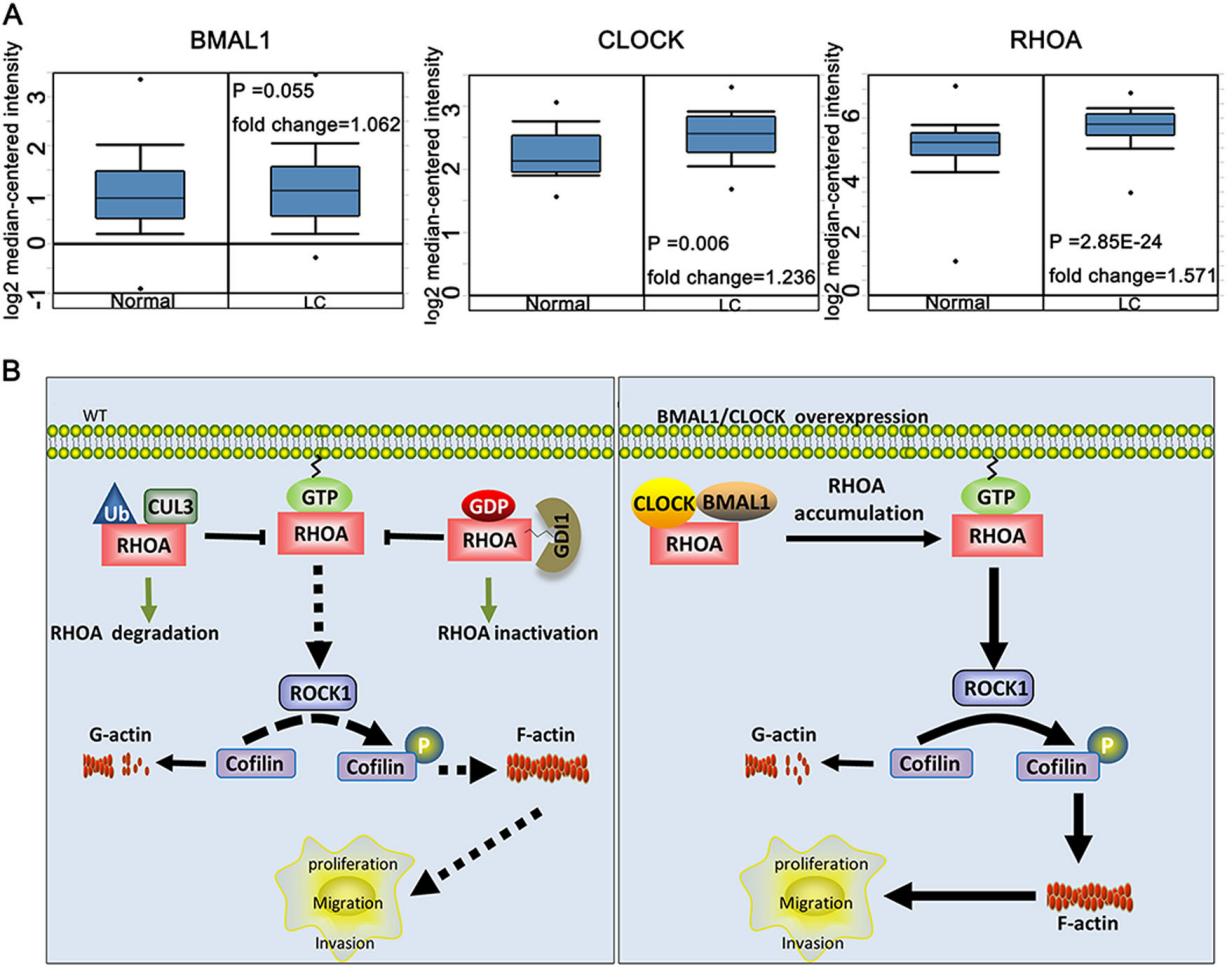
A schematic of the regulation of the RHOA-ROCK-CFL pathway by CLOCK and BMAL1 in tumor cells. **a** Analysis of BMAL1, CLOCK, and RHOA in liver cancer (*ONCOMINE* database). Box plots derived from gene expression data in *ONCOMINE* in normal and liver cancer tissue. The *p* value was set up at 0.01, and the fold change was defined as 2; LC liver cancer. **b** A schematic diagram depicting the regulatory effects of CLOCK and BMAL1 on F-actin/G-actin turnover and its role in cell proliferation, migration, and invasion

To investigate the clinical significance of CLOCK, BMAL1, and RHOA expression in tumor cells, we compared CLOCK, BMAL1, and RHOA expression between normal human liver and liver carcinoma tissues using the *Oncomine* database (http://www.oncomine.org). These data indicated that CLOCK, BMAL1, and RHOA were all upregulated in liver carcinoma when compared with the expression in normal liver tissues (Fig. 8a). The link identified in our research between BMAL1, CLOCK, and Rho GTPase activity presents an innovative therapeutic targeting strategy of Rho GTPase in cancer.

## Discussion

CLOCK and BMAL1 are traditionally considered to be two transcription factors that play core roles by forming CLOCK/BMAL1 heterodimers. These molecules initiate target gene transcription by binding to the promoter regions of the genes in mammals^28,29^. Nevertheless, our data provide novel mechanistic insight into circadian genes as a regulator to inhibit CUL3-mediated RHOA ubiquitination and suppress the binding of RhoGDIs to RHOA. These findings showed that CLOCK and BMAL1 can directly interact with the kinase pathway in addition to their traditional transcription activation function. Notably, we have provided evidence that, in this mechanism, CLOCK and BMAL1 still work together in one complex, and each of these two proteins positively affect RHOA activity. However, the question of whether their function depends on the heterodimer or whether they are able to function separately requires further investigation.

Our present results showed that overexpression of CLOCK or BMAL1 increased the amount of RHOA in HeLa cells (Fig. 3). Interestingly, BMAL1 overexpression more effectively regulated RHOA localization and expression than did CLOCK overexpression. A previous study^30^ showed that inflammation and necrosis factors are likely to induce the translocation of RHOA into the nucleus. However, the role of nuclear-located RHOA is still unclear.

Additionally, we found that CLOCK and BMAL1 affect the degradation of RHOA by affecting the binding to the E3 ligase, CUL3. The interaction between CUL3 and RHOA increased in CLOCK- or BMAL1-knockdown cells and decreased in CLOCK- or BMAL1-overexpressing cells, indicating that CLOCK and BMAL1 compete with CUL3 to bind RHOA and regulate F-actin dynamics.

Previous studies have shown that actin dynamics exhibit circadian regulation in some peripheral tissues in vivo and are proposed to regulate the activity of the core circadian clock complex in response to systemic cues^31,32^. In addition to timekeeping, clock genes have also been shown to have rhythmic functions associated with cytoskeleton regulation. Nathaniel et al.^33^ revealed that the cellular clock regulates cell migration through circadian control of cytoskeletal dynamics in fibroblasts, and Afroditi et al.^34^ demonstrated that rhythms in RHOA activity are controlled by clock-regulated transcription of puratrophin-1-like (Pura). However, our previous study indicated that the expression of CLOCK and BMAL1 demonstrate no circadian rhythmicity in tumor cells^35^.

In summary, our present research illustrates a novel insight into the circadian clock proteins, CLOCK and BMAL1, which can promote the proliferation, migration, and invasion of cancer cells by affecting the formation of F-actin. This presupposition was supported by two lines of evidence in our study. First, the overexpression of CLOCK and BMAL1 affected the levels of RHOA by blocking the degradation of RHOA. Second, the overexpression of CLOCK and BMAL1 affected the active state of RHOA by preventing the inactivation of RHOA (Fig. 8b). Considering that F-actin filament dynamics are involved in a variety of significant bioprocesses, our current research provides a new perspective on the roles of CLOCK and BMAL1 in tumor cells.

## Electronic supplementary material

Figure S1
