# Author Correction: CLOCK and BMAL1 stabilize and activate RHOA to promote F-actin formation in cancer cells

**DOI:** 10.1038/s12276-026-01722-2

**Published:** 2026-04-01

**Authors:** Teng-jiao Ma, Zhi-wei Zhang, Yi-lu Lu, Ying-ying Zhang, Da-chang Tao, Yun-qiang Liu, Yong-xin Ma

**Affiliations:** Department of Medical Genetics, State Key Laboratory of Biotherapy, West China Hospital and Collaborative Innovation Center, Sichuan University, 610041 Chengdu, China

Correction to: *Experimental & Molecular Medicine*

10.1038/s12276-018-0156-4, published online 04 October 2018

After online publication of this article, the authors noticed an error in the “Results” section.

In the article by Ma et al., during the preparation of Fig. 6, the authors introduced an error that resulted in the inclusion of an incorrect image in Fig. 6A. Unintentionally, the figure showed RHOA blottings obtained from CUL3 or BMAL1 knockdown cells, instead of an image from RHOA overexpression or knockdown cells. This error did not change the data or conclusions of the article in any way. The authors have hereby provided the corrected version of Fig. 6.

Incorrect figure 6
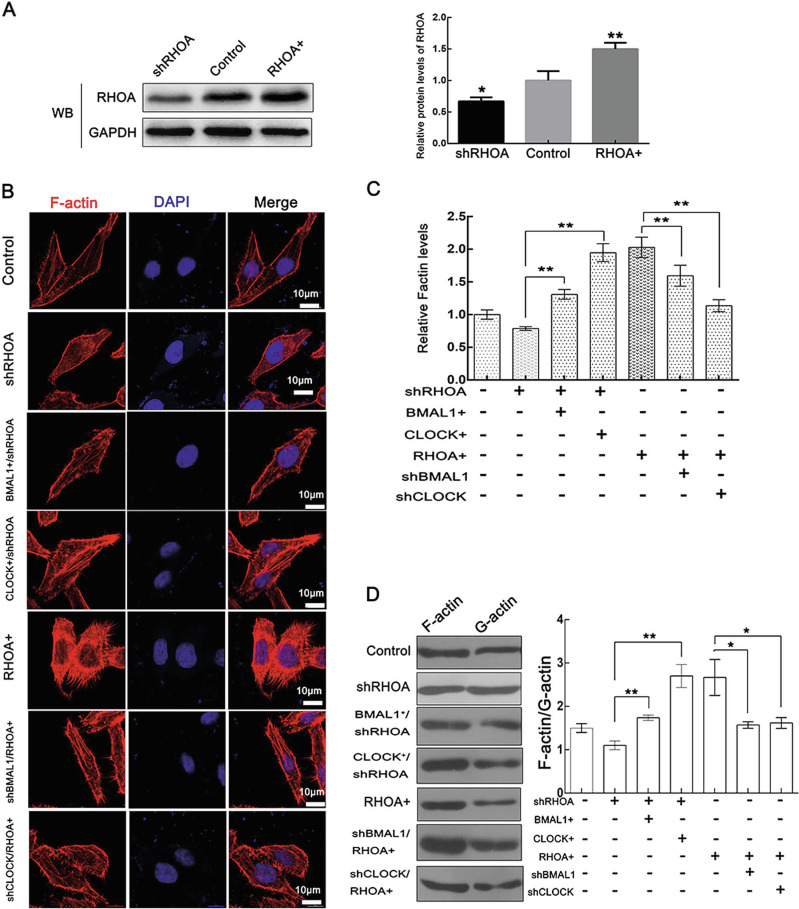


Correct figure 6
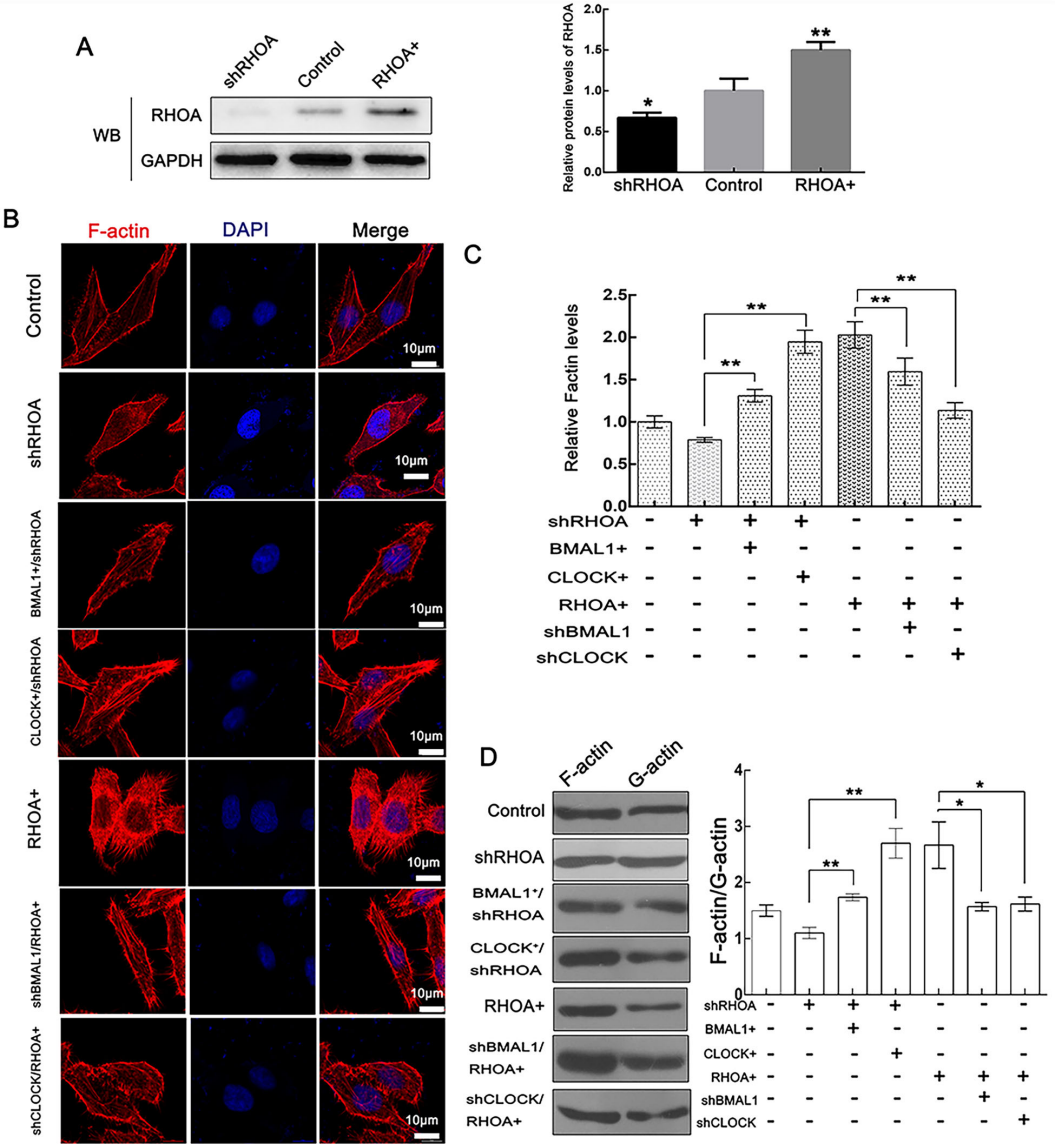


The authors apologize for any inconvenience caused.

The original article has been corrected.

