# Supplementary material for: CLOCK and BMAL1 stabilize and activate RHOA to promote F-actin formation in cancer cells
Source: Exp Mol Med. 2018 Oct 4;50(10):1–15. doi: 10.1038/s12276-018-0156-4 (PMC6172197; doi:10.1038/s12276-018-0156-4)
Supplement: Supplementary file 1 — Figure S1 [file 12276_2018_156_MOESM1_ESM.pdf]

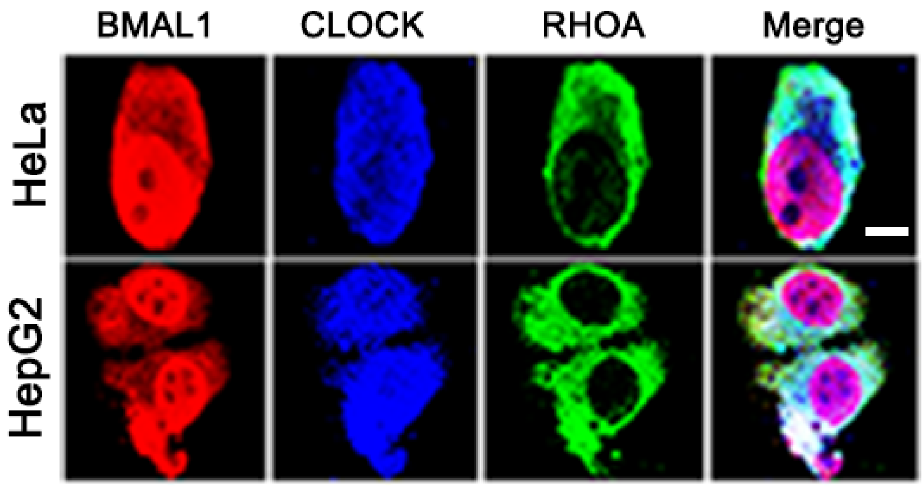

Figure S1. Immunofluorescent staining of endogenous BMAL1 (Red), CLOCK (Blue) and RHOA (Green) in HeLa cells. The scale bars represent 5μm.
